# Supplementary material for: Detecting stable adsorbates of (1S)-camphor on Cu(111) with Bayesian optimization
Source: Beilstein J Nanotechnol. 2020 Oct 19;11:1577–89. doi: 10.3762/bjnano.11.140 (PMC7590619; doi:10.3762/bjnano.11.140)
Supplement: File 1 — Camphor global minimum conformer, convergence of the 6D model, and coordinates of camphor. [file Beilstein_J_Nanotechnol-11-1577-s001.pdf]

BEILSTEIN JOURNAL OF NANOTECHNOLOGY

Supporting Information

for

Detecting stable adsorbates of (1*S*)-camphor on Cu(111) with Bayesian optimization

Jari Järvi, Patrick Rinke and Milica Todorović

Beilstein J. Nanotechnol. **2020,** *11,* 1577–1589. doi:10.3762/bjnano.11.140

Camphor global minimum conformer, convergence of the 6D model, and coordinates of camphor

License and Terms: This is a supporting information file under the terms of the Creative Commons Attribution License (https://creativecommons.org/licenses/by/4.0). Please note that the reuse, redistribution and reproduction in particular requires that the authors and source are credited.
The license is subject to the *Beilstein Journal of Nanotechnology* terms and conditions: (https://www.beilstein-journals.org/bjnano)

Camphor geometry in global minimum conformer search

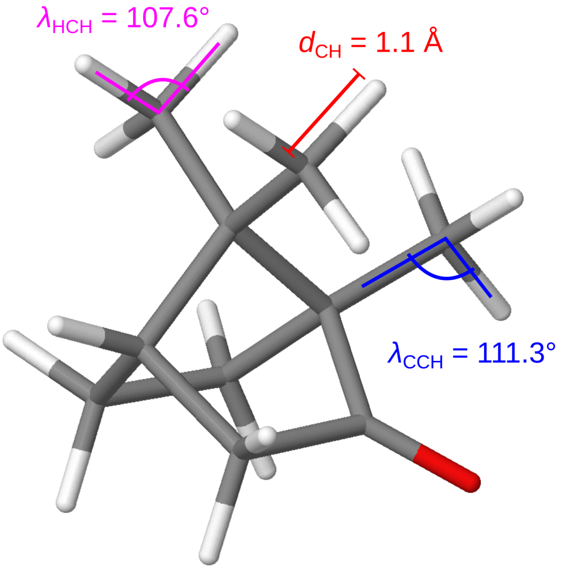

Figure S1: Ideal camphor geometry with a 120° periodic rotation of the methyl groups. The C–H bonds in the three methyl groups are normalized to their average relaxed bond length of d_{CH} = 1.1 Å, bond angle λ_{CCH} = 111.3° and the angle between H atoms λ_{HCH} = 107.6°.

Convergence of the 6D surrogate model

We evaluate the convergence of the surrogate model of the 6D PES with respect to the coordinates and adsoprtion energy of the identified local minima. For a reliable identification of all the minima, we converge the complete PES, not only the global minimum. In this process, we acquire new energy points with BOSS and DFT in batches. We follow the convergence of the 6D model by identifying the minima in the model after each batch. Before the local minima search, the acquired energy points are duplicated according to the translational symmetry in the orthogonal unit cell of the Cu(111) surface.

The minima are identified using the built-in local minima search functionality of BOSS. From each acquisition point, BOSS starts a minimizer, which traverses the landscape following the gradient to locate an energy minimum. The minimizers apply the limited-memory Broyden–Fletcher–Goldfarb–Shanno (L-BFGS) optimization algorithm. With this method, multiple minimizers typically end up in the same minimum. The duplicate minima are not removed, since they provide

information about the surrogate model, that is, how large region of the phase space a particular minimum occupies. Due to the varying number of acquisition points in different models, the number of employed minimizers varies between models. We analyzed the convergence of the 6D PES with three different models, constructed with 1218, 1380, and 1420 energy points (referred to as M1218, M1380, and M1420). In the local minima search, we consider the reliably identified minima to be the points, into which several minimizers have ended up after traversing the landscape.

We identify the minima by investigating the adsorption energy of each minimizer, sorted by energy (Figure S2). In this graph, the minima are shown as energy plateaus of varying lengths. The longer the plateau, the more minimizers have ended up in the particular minimum. The varying number of minimizers in each model shows as a horizontal shift of the graph between the three models. From this analysis, we conclude that the converged model is M1380. We then proceed by extracting the structures in the identified minima, verify them with full relaxation with DFT, and perform further analysis.

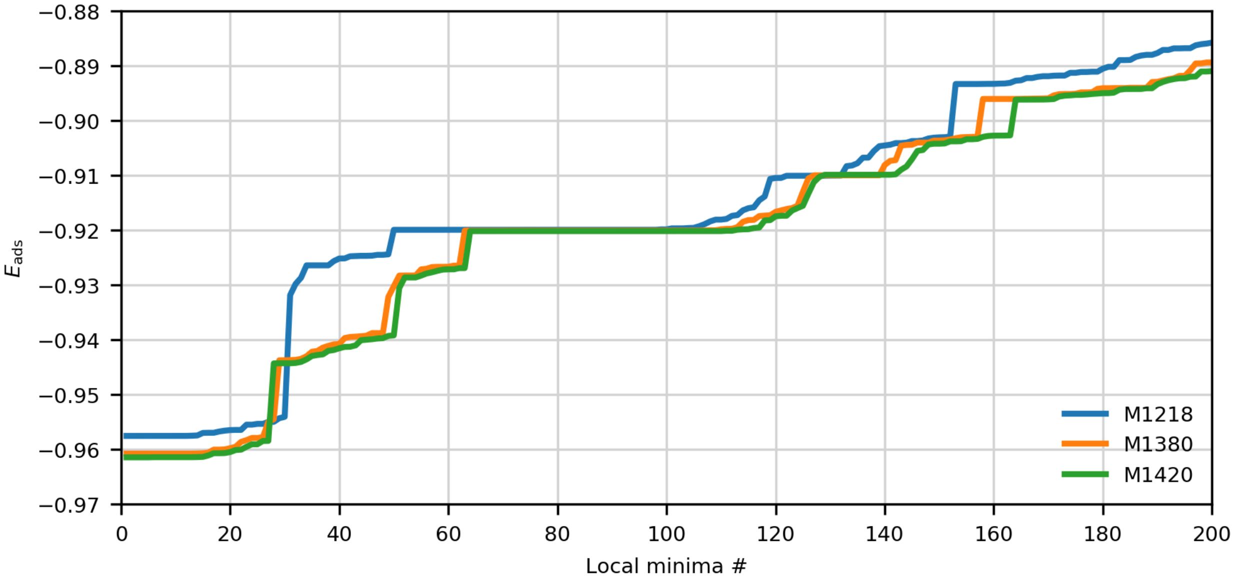

Figure S2: Adsorption energy (E_{ads}) of 200 lowest minima in three different surrogate models of the 6D PES, constructed with 1218, 1380, and 1420 energy points. The local minima are identified as points of identical energy, found by multiple minimizers, which are shown as energy plateaus in the graph.

Coordinates of camphor in the predicted and relaxed stable structures

We confirm the accuracy of the predicted stable structures, identified with Bayesian Optimization Structure Search (BOSS) in the minima of the 6D potential energy surface (PES), with a full relaxation with density-functional theory (DFT). Here, we compare the three translational (x, y, z) and three rotational (α, β, γ) coordinates of camphor in the minima of the 6D PES (Table S1) to the corresponding coordinates after full relaxation of the structures (Table S2). We then evaluate the structural changes in the relaxation via difference of the coordinates before and after the relaxation (Table S3).

Table S1: Coordinates of camphor in the identified stable structures (i.e. structures with building block approximation), which correspond to the minima of the 6D PES.

	x (frac.)	y (frac.)	x (Å)	y (Å)	z (Å)	α (deg)	β (deg)	γ (deg)
Ox1	−0.271	−0.301	−0.695	−1.337	4.174	12.7	5.8	−153.6
Ox2	0.206	0.326	0.528	1.451	4.454	−85.7	161.6	2.2
Ox3	−0.393	−0.018	−1.009	−0.078	4.404	4.8	25.5	173.7
Ox4	0.183	0.326	0.471	1.451	4.603	31.5	3.8	−102.4
Ox5	0.004	−0.005	0.011	−0.023	4.973	58.6	46.8	−58.4
Hy1	−0.001	0.000	−0.003	0.000	5.000	−111.1	32.0	−175.0
Hy2	−0.463	−0.430	−1.190	−1.912	4.071	−98.4	−90.9	−81.3
Hy3	0.000	0.000	−0.001	0.000	5.023	178.8	33.8	−34.9

Table S2: Coordinates of camphor in the stable structures after full relaxation with DFT. The rotational coordinates of structure Hy2 are not uniquely defined (denoted with ***) and therefore omitted here. A visual comparison of structure Hy2 before and after the relaxation is shown in Figure S3.

	x (frac.)	y (frac.)	x (Å)	y (Å)	z (Å)	α (deg)	β (deg)	γ (deg)
Ox1	−0.281	−0.306	−0.721	−1.362	4.127	12.9	4.2	−152.3
Ox2	0.198	0.374	0.508	1.662	4.379	−93.5	169.0	−0.4
Ox3	−0.411	−0.020	−1.055	−0.088	4.130	9.1	15.6	178.3
Ox4	0.292	0.296	0.749	1.318	4.424	38.8	−4.3	−96.6
Ox5	0.018	−0.017	0.045	−0.077	4.778	56.8	44.8	−58.6
Hy1	0.221	0.023	0.567	0.101	4.339	−89.8	20.9	−171.9
Hy2	−0.490	−0.474	−1.257	−2.109	3.964	* * *	* * *	* * *
Hy3	−0.001	−0.001	−0.002	−0.005	5.030	178.8	34.0	−34.8

Table S3: Structural changes in the identified stable adsorbates, comparing the predicted structures (i.e., structures with building block approximation) to the structures after full relaxation with DFT. Change in the location and orientation of camphor is given with respect to translational and rotational coordinates, (Δx, Δy, Δz) and ($\Delta\alpha$, $\Delta\beta$, $\Delta\gamma$), respectively. Change in the internal geometry of camphor is given as root-mean-square deviation of the atomic positions (δ^A) and the mean deviation of bond lengths (δ^B). Changes in the rotational coordinates of structure Hy2 are not uniquely defined (denoted with ***) and therefore omitted here. A visual comparison of structure Hy2 before and after the relaxation is shown in Figure S3.

	Δx (Å)	Δy (Å)	Δz (Å)	$\Delta\alpha$ (deg)	$\Delta\beta$ (deg)	$\Delta\gamma$ (deg)	δ^A (Å)	δ^B (Å)
Ox1	−0.026	−0.025	−0.047	+0.2	−1.6	+1.3	0.033	0.0036
Ox2	−0.020	+0.211	−0.074	−7.8	+7.4	−2.6	0.136	0.0040
Ox3	−0.046	−0.009	−0.274	+4.3	−9.9	+4.6	0.142	0.0036
Ox4	+0.278	−0.133	−0.178	+7.3	−8.1	+5.8	0.180	0.0041
Ox5	+0.035	−0.055	−0.195	−1.8	−2.0	−0.2	0.072	0.0036
Hy1	+0.570	+0.101	−0.661	+21.3	−11.1	+3.1	0.353	0.0020
Hy2	−0.068	−0.198	−0.108	* * *	* * *	* * *	0.136	0.0025
Hy3	−0.001	−0.005	+0.007	−0.0	+0.2	+0.1	0.010	0.0015

We applied BOSS to solve the rotational coordinates of the relaxed structures. With a 3D search, BOSS acquired different molecular orientations and identified the rotation $R(\alpha, \beta, \gamma)$ that produces a matching orientation with the relaxed structure.

With structure Hy2, the rotational coordinates are not uniquely defined. Due to the β rotation angle of ca. $-90°$ in Hy2, the α and γ rotations are coupled, such that identical orientations can be produced with various different values of α and γ. We have therefore omitted the rotational coordinates of the relaxed structure Hy2 in Table S2 and Table S3 (denoted with ***). Nevertheless, we verified structure Hy2 with visual comparison before and after the relaxation (Figure S3), which clearly shows that the structural changes in the relaxation are minimal.

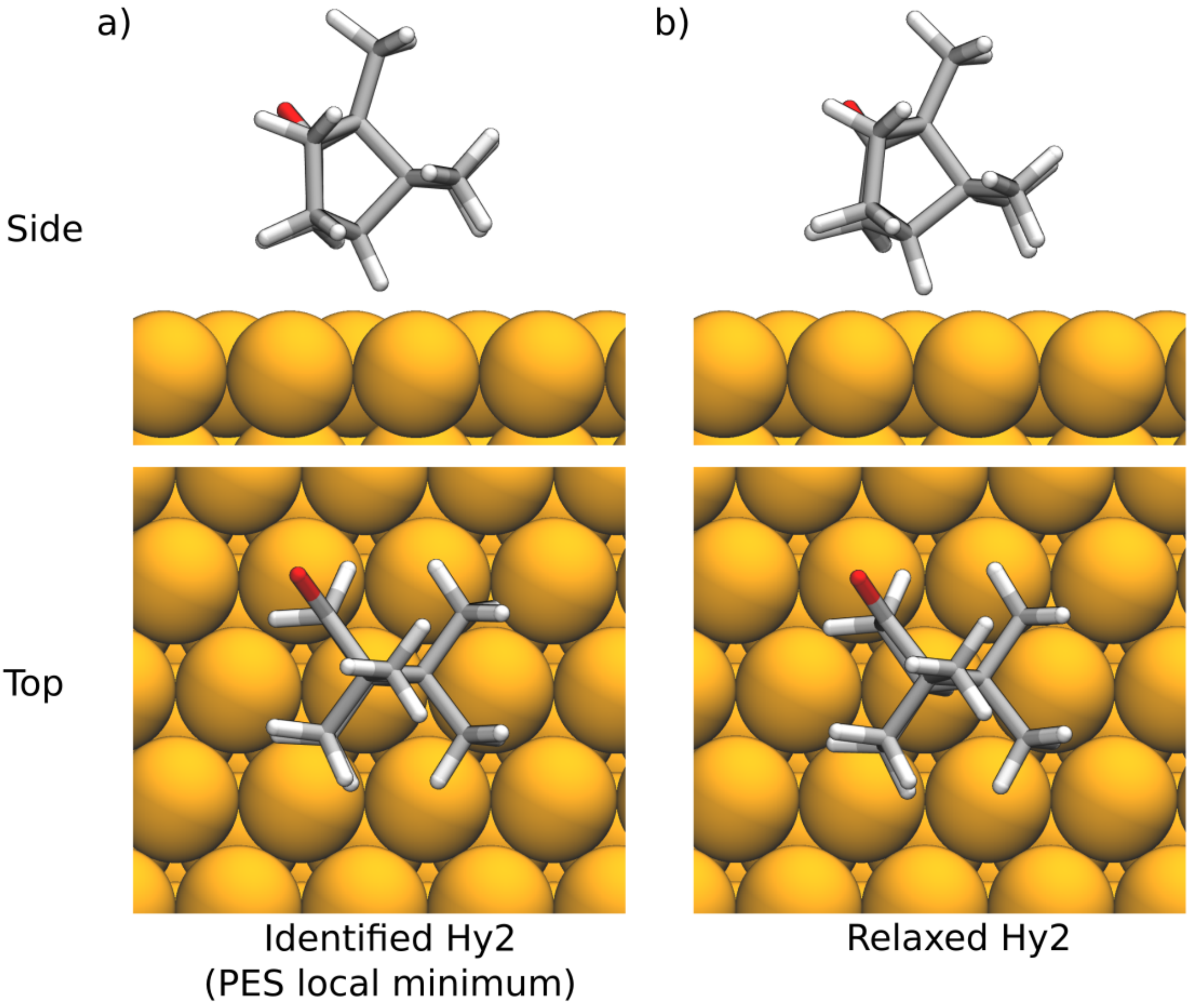

Figure S3: a) Stable structure Hy2, identified in the local minimum of the 6D PES (i.e. with building block approximation), and b) after full relaxation with DFT.
